# RSV-NTHi Co-Infection Skews Host Immunity by Suppressing Type I IFN Responses and Enhancing Pro-Inflammatory Responses

**DOI:** 10.3390/pathogens15030240

**Published:** 2026-02-24

**Authors:** Zhinian Zhou, Justin W. Brennan, Ann Lindley Gill, Christopher S. Anderson, Brian M. Ward, Gloria Pryhuber, Thomas J. Mariani, Steven R. Gill, Yan Sun

**Affiliations:** 1 Department of Immunology and Microbiology, University of Rochester Medical Center, Rochester, NY 14642, USA; zhinian_zhou@urmc.rochester.edu (Z.Z.); justin_brennan@urmc.rochester.edu (J.W.B.); ann_gill@urmc.rochester.edu (A.L.G.); brian_ward@urmc.rochester.edu (B.M.W.); steven_gill@urmc.rochester.edu (S.R.G.); 2 Department of Pediatrics and Center for Children’s Health Research, University of Rochester, Rochester, NY 14642, USA; christopher_anderson@urmc.rochester.edu (C.S.A.); gloria_pryhuber@urmc.rochester.edu (G.P.); tom_mariani@urmc.rochester.edu (T.J.M.)

**Keywords:** RSV, NTHi, DVGs, type I and type III IFNs, inflammation, human precision-cut lung slices

## Abstract

Respiratory syncytial virus (RSV) is a leading cause of lower respiratory tract disease in young children, yet determinants of disease severity in otherwise healthy infants remain poorly understood. RSV disease severity has been linked to temporal differences in airway epithelial type I/III IFN and inflammatory responses, with defective viral genomes (DVGs) acting as potent inducers of type I/III IFNs. Additionally, an increased abundance of nontypeable *Haemophilus influenzae* (NTHi) is associated with more severe RSV disease, although the mechanisms underlying this association and the impacts of NTHi on DVG replication and DVG-driven host responses remain unclear. To address this knowledge gap, we modeled a clinically relevant but underexplored scenario of simultaneous transmission by performing concurrent RSV–NTHi co-infection experiments. Co-infection reduced titers of RSV but not of NTHi. Mechanistically, extracellular NTHi inhibited RSV particle binding to host cells. Using A549 cells and human precision-cut lung slices, we found that NTHi alone was a weak inducer of type I/III IFNs but a strong inducer of pro-inflammatory cytokines. Accordingly, RSV–NTHi co-infection suppressed DVG replication and DVG-driven IFN responses while enhancing inflammatory signaling, potentially driven by increased cell-associated NTHi. Together, these findings provide a mechanistic basis for how NTHi exacerbates RSV disease severity through dysregulated host immune responses rather than increased viral burden.

## 1. Introduction

RSV infects approximately 70% of newborn infants annually and is a leading cause of lower respiratory tract disease in young children, resulting in ~100,000 hospitalizations in the United States and nearly 3 million hospitalizations worldwide each year. The clinical manifestations of RSV infection range from mild upper respiratory symptoms to severe lower respiratory tract disease, including bronchiolitis and pneumonia. Known risk factors for severe RSV infection include young age, prematurity, chronic lung disease, congenital heart disease, and environmental factors such as exposure to tobacco smoke [1]. Despite these recognized risk factors, the majority of severe RSV cases occur in otherwise healthy infants, highlighting a critical gap in our understanding of the host mechanisms that determine the severity of clinical outcomes during RSV infection. We performed high-throughput genomic analysis on longitudinal samples from RSV-infected infants and identified that numerous lymphotropic and immune cell chemokines correlate with disease severity [2]. Importantly, mildly and severely ill patients fundamentally differ in their airway epithelial cell type I/III interferon (IFN) responses, with mildly ill subjects displaying evidence of a robust, acute induction of IFN ligand production and signaling compared to severely ill patients [2].

RSV is prone to generating defective viral genomes (DVGs), which are truncated forms of viral genomes. They are the primary triggers of type I/III IFN responses during RSV infection, more potent than full-length viral genomes (FL-gRSV), and have been shown to critically impact RSV pathogenesis and disease severity. RSV encodes strong IFN antagonists such as the NS1 and NS2 proteins [3], resulting in minimal IFN responses at the early stage of viral infection when few, or no, DVGs are present in the viral community (RSV with a low DVG content, referred to as RSV LD). However, RSV enriched with DVGs (RSV with a high DVG content, referred to as RSV HD) overcomes this IFN inhibition and stimulates rapid and robust expression of type I/III IFNs and ISGs both in vitro and in mice [4]. In patients, the detection of DVGs in the respiratory secretions of children infected with RSV positively correlates with the expression of genes with antiviral activity [4]. Importantly, the kinetics of RSV DVG production in adult patients is associated with disease severity with late/prolonged DVG detection, correlating with higher viral load, enhanced inflammation, and worse symptom scores [5]. Interestingly, in human precision-cut lung slices (hPCLSs), RSV LD infection in certain donors results in delayed DVG accumulation accompanied by delayed IFN responses and an overall higher viral load [4]. These findings closely align with our previous report that severe disease is associated with delayed IFN responses and subsequent enhancement of pro-inflammatory cytokine expression [2], with DVGs likely contributing to these host immune dynamics.

Beyond host immune responses and the role of DVGs, recent work on the respiratory microbiome has illustrated the importance of viewing the respiratory microbiota as a complex community that cooperatively interacts with RSV and potentially modulates the disease response [6,7,8,9,10]. Several studies, including our own, have reported that an increased relative abundance of *Haemophilus influenzae*—particularly nontypeable *H. influenzae* (NTHi)—in the nasal microbiome is associated with more severe RSV disease [11,12,13,14]. NTHi is a common opportunistic bacterium that frequently colonizes the upper respiratory tract of healthy individuals; however, it is also a leading cause of noninvasive mucosal infections such as otitis media, sinusitis, and conjunctivitis [15]. Despite these associations, the mechanisms by which NTHi enhances RSV disease severity remain unclear. The reported effects of NTHi on RSV infection are conflicting: one study demonstrated that NTHi infection protects host cells from subsequent RSV infection, whereas another showed that pre-treatment with heat-killed NTHi increases the susceptibility of airway epithelial cells to RSV infection and pro-inflammatory responses [7,16]. In contrast, prior RSV infection has been consistently shown to enhance NTHi colonization or abundance in vitro and in animal models [17,18,19,20]. To date, how RSV–NTHi co-infection differentially affects each pathogen and how these interactions directly shape host antiviral IFN responses and pro-inflammatory signaling are not fully understood.

A critical variable proposed in prior studies is the order of infection of the two pathogens, with most studies investigating sequential infection. However, in hospitalized infants who test positive for both RSV and NTHi, it remains unclear whether NTHi colonization precedes RSV infection, follows it, or whether both pathogens are acquired at the same time. To model a less-studied but clinically relevant scenario in which viral and bacterial pathogens are co-transmitted and establish infection simultaneously, we performed concurrent RSV and NTHi infection experiments in this study. We found that co-infection reduced RSV titers but did not significantly alter NTHi titers in the supernatants from infected A549 cells; also, RSV suppression was dependent on the abundance of extracellular NTHi. Extracellular NTHi, regardless of live or dead status, inhibited RSV particle binding to host cells when present at sufficient levels. Regarding host responses, NTHi was a weak inducer of type I and III IFNs but a strong inducer of pro-inflammatory cytokines compared to RSV. Simultaneous RSV–NTHi co-infection in both A549 cells and hPCLS tissues suppressed the innate IFN responses triggered by RSV DVGs while further enhancing pro-inflammatory signaling. Consistent with previous studies, RSV infection increased the cell-associated NTHi abundance, which may contribute to the elevated inflammation observed during co-infection. Together, our data provided a molecular basis for how NTHi exacerbates RSV disease severity.

## 2. Materials and Methods

### 2.1. Cell and hPCLS Maintenance

A549, interferon alpha-receptor knockout A549 cells (IFNAR KO), HEp2, and Vero cells were maintained in tissue culture medium (TCM) at 37 °C and 5% CO_2_. The TCM for culturing A549 and HEp2 cells contained Dulbecco’s modified Eagle’s medium (DMEM) with 10% heat-inactivated fetal bovine serum (HI-FBS), 2 mM L-Glutamine, 1 mM sodium pyruvate, and 50 μg/mL gentamicin. One day before infection, medium was changed to the same TCM formulation without any antibiotics. The hPCLSs from pediatric donors (age < 5 yrs, one donor per repeat), obtained from LungMAP (URMC), were processed according to protocols published elsewhere [21,22] and stored in liquid nitrogen. The hPCLSs were maintained at an air–liquid interface culturing system using transwell inserts with a 0.4 μm permeable polyester membrane (Corning) in 6-well plates. The apical chambers of the transwells were pretreated with 100 μL of rat-tail collagen (BD Biosciences CA, USA, #354236; 1:100 diluted in 100% EtOH), followed by air-drying for 1 h. hPCLSs (one slice per well) were then placed in the apical chamber and washed twice with DPBS, followed by incubation at 37 °C and 5% CO_2_ for 30 min to allow for attachment. A volume of 900 μL of PCLS media (500 mL DMEM, 10% FBS, 1 mM sodium pyruvate, 1X non-essential amino acids, 15 mM HEPES buffer) with antibiotics (50,000 U penicillin/streptavidin, 125 μg of amphotericin, 40 μg of gentamicin) was then added to the basal chamber. After 24 h of incubation at 37 °C, the basal medium was replaced with 900 μL of PCLS media without antibiotics for an additional 48 h, after which infection was performed.

### 2.2. RSV Preparation and Titration

All RSV stocks were maintained and titrated in HEp2 cells. For generation of the RSV LD working stock, HEp2 cells were infected with RSV (A2 strain obtained from ATCC, passage 3) at an MOI of 0.001–0.002 in infection medium (TCM supplemented with 2% FBS, Gentamicin free) for 5–6 days. RSV HD working stocks were generated as previously described [22]. Briefly, RSV A2 was passaged in HEp2 cells infected at an MOI of 5–10 in infection medium for 3 days. Additional HEp2 cells were then inoculated with the preceding passage at MOI 5, and each MOI 5 passage proceeded for 3 days. DVG enrichment proceeded for four total passages. Before performing infection experiments, the LD/HD virus stocks were characterized using DVG-specific RT-PCR to confirm the depletion and enrichment, respectively, of DVGs; then, qRT-PCR measuring the host responses after infection was performed, as described previously [23]. To generate RSV-GFP (rg224 [24]) and RSV-tdTomato (rrRSV131 [25]) working stocks, HEp2 cells were infected at an MOI of 0.5 and harvested 1–2 days post-infection when ~100% of cells expressed GFP or tdTomato. For harvesting, cells were scraped and the cells/supernatants were centrifugated at 600× *g* for 5 min at 4 °C. To release cell-associated virus, the cell pellet was quick-frozen/thawed three times and centrifugated at 600× *g* for 5 min at 4 °C. Clarified virus-containing supernatants were combined and aliquoted, quick-frozen, and stored at −80 °C. RSV LD/HD stocks were titered via a Median Tissue Culture Infectious Dose (TCID50) assay. For titration of RSV-GFP and RSV-tdTomato stocks, both stocks were serially diluted 5-fold and fluorescence forming units (FFUs) were counted 1 day post-infection.

### 2.3. NTHi Preparation and Titration

The NTHi growth culture was brain heart infusion (BHI) supplemented with 1/100 diluted Hemin stock solution and 100 μg/mL of NAD+ (10 mg Hemin was dissolved in 10 mL 0.01 N NaOH solution to make the Hemin stock). To prepare NTHi (86-028NP) stocks, NTHi was grown in 500 mL of supplemented BHI, shaken at 37 °C until the OD reached approximately 0.8. NTHi was centrifugated at 6000 rpm for 20 min at 4 °C and washed twice with 50 mL of PBS. After washing, NTHi was resuspended in 4 mL PBS with 25% Glycerol, aliquoted (100 μL/vial), and stored at −80 °C. The original NTHi-GFP (86-028NP) was obtained from Dr. Steven Gill’s lab. To prepare the NTHi-GFP stock, seed bacteria were plated on chocolate agar plates and single colonies were isolated by colony isolation streaking. Selected colonies were inoculated in 5 mL of BHI culture media supplemented with 200 μg/mL of spectinomycin. After overnight growth in the shaker (250 rpm, 37 °C) in the dark, the culture was mixed with Glycerol at a 1:1 ratio, aliquoted, and stored at −80 °C. The titer of NTHi stocks was determined by 10-fold serial dilution and plate-counting on a chocolate agar plate incubated overnight at 37 °C. To determine the percentage of NTHi-GFP fluorescing, the plate was additionally read using a Bio-Rad plate reader (channel: alexa 488), and colonies fluorescing green were counted. Compared to the total CFU count obtained from the chocolate agar plate, 89.5% of our NTHi-GFP colonies showed a green fluorescent signal. Subsequent experiments were based on total CFU count. NTHi 86-028NP mCherry pKM1.1 (NTHI-mCherry) was maintained/titered similarly to the above protocol. To prepare NTHi for infection, NTHi stocks were diluted in infection TCM without antibiotics at designated MOIs. For preparing heat-killed NTHi, NTHi was incubated at 65 °C for 30 min (vortexed every 10 min), and placed on ice for ten minutes.

### 2.4. NTHi-RSV Co-Infection in A549 Cells and in hPCLS

A549 cells were co-infected with RSV and NTHi simultaneously. Specifically, RSV-GFP or RSV LD/HD (MOI of 0.5) and NTHi (MOI of 5) were mixed in the infection medium. The mixtures were incubated at 37 °C for 1 h (mixed by gently inverting every 20 min). After incubation, the cells were inoculated for 2 h at 37 °C. The inoculum was removed and cells were washed 3 times with PBS; then, 1 ml of fresh infection medium was added and cells were incubated at 37 °C until harvest. Infected cells were harvested for flow cytometry, RNA extraction, or bacterial DNA purification. Additionally, at the time of harvest, 100 μL of the supernatants was directly quick-frozen for RSV titration, whereas 100 μL of the supernatants was mixed with 25 μL of Glycerol for bacterial titration. If cell death was observed, especially at a later time (48 hpi), supernatants were first centrifuged at 6000 rpm 5 min at 4 °C to clarify dead cells. Supernatants were then processed similarly as stated above.

For hPCLSs, 1 × 10^6^ PFU/slice of RSV (LD or HD) was mixed with 1 × 10^7^ CFU/slice of NTHi. The mixture was incubated at 37 °C for 1 h (mixed by gently inverting every 20 min) and added dropwise to hPCLSs on the apical chamber and incubated at 37 °C with 5% CO_2_ for 2 h (with gentle shaking every 20 min). The inoculum was then removed and hPCLSs were washed twice with DPBS. Basal chamber medium was refreshed daily and infected tissues were harvested at designated time points post-infection by homogenizing tissues in 1 mL of TRizol for total RNA extraction. For visualizing the effect of NTHi on RSV infection in the hPCLS, the slices were similarly co-infected with RSV-tdTomato and NTHi. At 24 hpi, images were taken with a Leica DMIRB inverted microscope at 50× magnification. At 48 hpi, images were taken with a Nikon Eclipse 80i Upright Fluorescence Phase Contrast Microscope (Nikon, Belmont, CA, USA) at 100× magnification.

### 2.5. RSV Binding Assay

Similar to the previous simultaneous in vitro co-infections, cells were incubated with RSV (MOI = 5) and viable NTHi (MOI = 50) or HK NTHi (MOI = 50/100/200) at 4 °C for 1 h. RSV/NTHi were mixed and incubated at 37 °C for 1 h and then chilled on ice for 10 min. Prior to infection, A549 cells were incubated at 4 °C for 30 min. After 1 h incubation, cells were washed with 1 ml of ice-cold PBS 3–5 times. For the experimental groups, cells were directly lysed in TRIzol for RNA extraction. As a negative control, cells were treated with 0.05% Trypsin-EDTA to dissociate viral particles from attaching to the cells after PBS washing. Cells were centrifuged at 600× *g* for 5 min at 4 °C, washed 3 times with PBS, and lysed in TRIzol for total RNA extraction.

### 2.6. NTHi DNA Purification

To quantify the cell-associated NTHi, A549 cells were firstly infected with RSV LD or HD (MOI = 0.5) for 6 h. After extensive washing, cells were inoculated with NTHi (MOI = 5) for 1 h, extensively washed, and then harvested at 40 h post-NTHi inoculation. At harvest, the mock or infected A549 cells were washed 3 times with PBS, followed by scraping and centrifugation at 600× *g* for 5 min at 4 °C to obtain cell pellets, where the genomic DNA of cell-associated NTHi was extracted according to the manufacturer’s instructions (DNeasy Blood and Tissue Kit, Qiagen, Germantown, MD, USA, #69504). Briefly, the NTHi-associated cell pellet was first lysed in 180 µL TE buffer with 10 μL of 100 mg/mL lysozyme and 5 μL of 10 mg/mL RNaseA at 37 °C for 30 min, and then further lysed in 25 µL of Proteinase K and 200 µL buffer AL from the kit at 70 °C for 30 min, followed by mixing with 200 µL of 100% EtOH. Then, the DNA was purified using a DNeasy Mini spin column.

### 2.7. RNA Extraction, Reverse Transcription, PCR, and qPCR

Upon harvest, the in vitro cell lines or ex vivo hPCLSs were directly lysed in 500 μL of TRIzol or homogenized in 1 mL of TRIzol on ice, respectively. The RNA was purified from cell/hPCLS lysate according to the manufacturer’s instructions. A volume of 250–1000 ng of total RNA was reverse-transcribed using a high-capacity RNA-to-cDNA kit (Applied Biosystems, Bedford, MA, USA) at 37 °C for 1 h. cDNA was diluted in nuclease-free water (1:20 or 1:40) for qPCR. The expression of host genes, RSV G, and NTHi HPD were examined with qPCR using PowerTrack™ SYBR Green Master Mix (Applied Biosystems). The relative copy numbers for cellular and viral genes were normalized by housekeeping gene GAPDH. The NTHi HPD absolute Ct values were converted to CFU/mL via a standard curve. To generate the standard curve, NTHi was grown in 8 mL of supplemented BHI culture overnight. Then, 1 mL of the culture was taken from either the undiluted or serially diluted sample (1/10, 1/100, and 1/1000). NTHi was pelleted by centrifugation at 6000 rpm for 20 min at 4 °C and DNA was purified as described above. Ct values of the NTHi HPD gene were measured by qPCR. In parallel, leftover undiluted and diluted samples were titered for plate-counting on chocolate agar plates. Finally, the log (CFU/mL) derived from the titration was plotted against the absolute HPD Ct value to obtain the standard curve. For DVG-specific reverse transcription, 1 μg of RNA was reverse-transcribed using RSV DI1-F primer (50 μM) and SuperScript III reverse transcriptase (Invitrogen, Waltham, MA, USA) followed by RNaseH digestion. For DVG-specific PCR, 2–4 μL of cDNA was mixed with 2.5 μL of 10X buffer, 2.5 μL of 50 mM MgSO_4_, 2.0 μL of 10 mM dNTPs, 1.0 μLof each of the 10 μM RSV DI1-F primer and 10 μM gRSV DI-R primer, 0.25 μL of 5 U/μL Platinum *Taq* polymerase (Invitrogen), and water to reach a total volume of 25 μL. The program used for PCR was 95 °C for 10 min and 34× amplification cycles (95 °C for 30 s, 55 °C for 30 s, and 72 °C for 90 s), and then hold at 72 °C for 5 min. Sequences of all primers used for RT, PCR, and qPCR are listed in Table 1.

### 2.8. Flow Cytometry

Upon harvest, cells were washed with PBS once, followed by dissociation with 0.05% Trypsin-EDTA. The detached cells were collected in FACS buffer (PBS with 1% FBS) to reach a total volume of 1 mL/well, followed by centrifugation at 600× *g* for 5 min at 4 °C, and the cell pellet was fixed by resuspending and incubating in 200 μL 2% PFA for 15 min at room temperature. Fixed cells were then washed twice with 200 μL FACS buffer and centrifugated using the same settings. Finally, the cells were resuspended in 200 μL FACS buffer for flow cytometry (LSRII Blue B 515 20 A for 30,000 events) and data were analyzed in Flowjo V10.

### 2.9. Statical Analysis

The details of the analysis are indicated in each respective figure legend. Statistical analyses were performed in GraphPad Prism version 8 (GraphPad Software).

## 3. Results

### 3.1. Co-Infection of RSV-NTHi Reduced RSV but Not NTHi Infection

**Figure 1:**
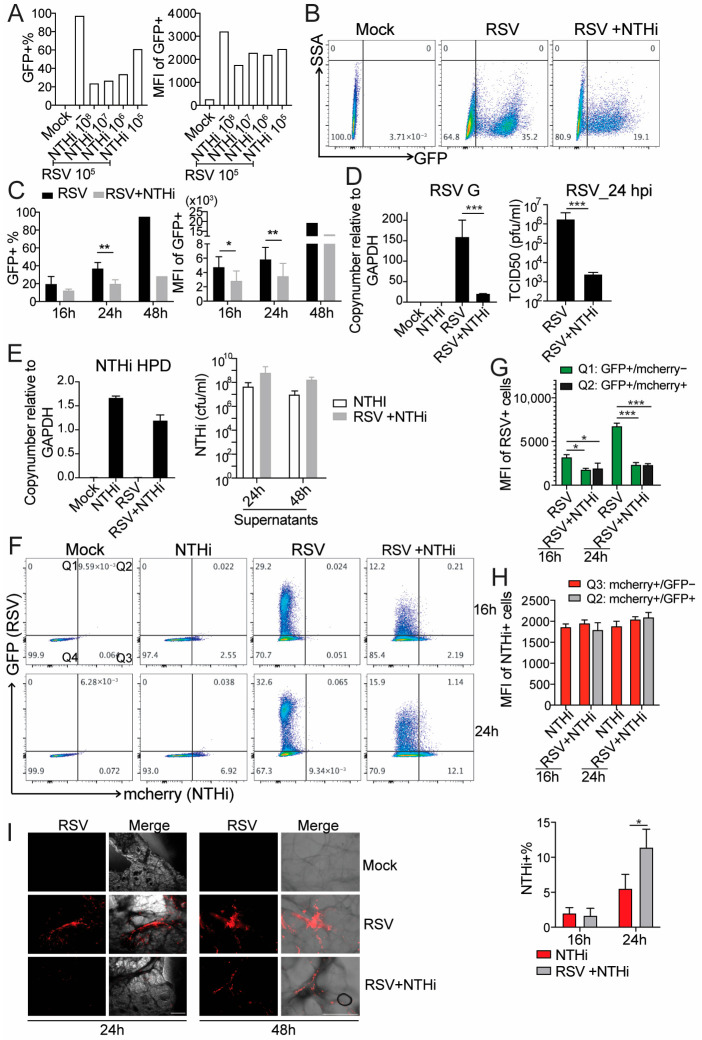
Co-infection of RSV-NTHi reduced RSV but not NTHi infection. (**A**) A549 cells were co-infected with RSV-GFP (MOI 0.5) and NTHi at varying RSV:NTHi ratios (1 × 10^8^ for 1:1000, 1 × 10^7^ for 1:100, 1 × 10^6^ for 1:10, 1 × 10^5^ for 1:1). Flow cytometric analysis was performed at 24 h post-infection to assess the GFP+% and MFI of GFP+ cells (N = 1). (**B**,**C**) A549 cells were co-infected with RSV-GFP and NTHi at a ratio of 1:10. Cells were analyzed by flow cytometry at 16, 24, 48 h post-infection. The representative flow plots at 24 hpi are shown in (**B**) and the quantification of all analyzed time points are graphed in (**C**). N = 3 for 16 and 24 h post-infection and N = 1 for 48 h post-infection. * *p* < 0.05, ** *p* < 0.01 according to two-way ANOVA. (**D**,**E**) A549 cells were co-infected similarly at an RSV:NTHi ratio of 1:10. Infected cells were collected to examine the expression of RSV G (**D**) and NTHi HPD (**E**) genes at 24 hpi. Supernatants were additionally collected to titrate RSV particles (**D**) and NTHi (**E**) at indicated time points. *** *p* < 0.001 according to unpaired *t*-test. (**F**–**H**) A549 cells were infected with RSV-GFP and NTHi-mcherry at a ratio of 1:10, similarly to as described above. Cells were collected for flow cytometry at 16 and 24 h post-infection. Representative flow plots are shown in (**F**). MFI for indicated RSV-related population are graphed in (**G**). MFI and percentage of indicated NTHi-related population are graphed in (**H**). * *p* < 0.05, *** *p* < 0.001 according to two-way ANOVA. (**I**) hPCLSs were co-infected with RSV-tdTomato and/or NTHi at 10^6^ PFU and 10^7^ CFU, respectively. Pictures of hPCLSs were taken during culture at 24 and 48 hpi, at 50× and 100× magnification, respectively. For pictures taken at 24 hpi, scale bar = 500 μm. For picture taken at 48 hpi, scale bar = 200 μm. N = 1 donor. The rest of experiments illustrated in this figure were repeated 3–4 times.

To first determine whether RSV–NTHi co-infection suppresses RSV infection, A549 cells were simultaneously co-infected with GFP-expressing RSV at an MOI of 0.5 (10^5^ PFU) and increasing amounts of NTHi. Flow cytometric analysis revealed a dose-dependent reduction in both the percentage of GFP-positive cells and the mean fluorescence intensity (MFI) of GFP-positive cells during co-infection (Figure 1A). An obvious reduction was observed starting from an RSV:NTHi ratio of 1:10 (10^5^ PFU RSV [MOI = 0.5] and 10^6^ CFU NTHi [MOI = 5]). Based on these results, we selected a 1:10 virus-to-bacterium ratio with an RSV MOI of 0.5 for subsequent experiments. To examine the kinetics of RSV suppression by NTHi, we performed the same co-infection at the selected dose and harvested samples at 16, 24, and 48 h post-infection (hpi). A trend toward reduced RSV levels was observed at 16 hpi, which became more pronounced at 24 and 48 hpi (Figure 1B,C).

To examine the effect of co-infection on NTHi levels, we performed the same infection experiment and then extracted the total RNA from infected cells, which we then followed with quantification of viral and bacterial gene expression via qPCR. Additionally, infectious virus or bacterial levels in the supernatant were determined as TCID_50_/mL and CFU/mL values, respectively. Consistent with our previous observations, both RSV G gene expression and RSV titers were reduced in the presence of NTHi compared to those with RSV infection alone (Figure 1D). In contrast, the NTHi HPD gene expression and bacterial titers were not significantly different between the single and co-infected cells. Notably, we consistently observed a trend toward higher NTHi levels in the supernatants during co-infection compared with NTHi-only infection (Figure 1E).

To determine which cell populations exhibited reduced RSV infection during co-infection, A549 cells were co-infected with RSV-GFP and mCherry-expressing NTHi and analyzed by flow cytometry. The MFI of GFP was significantly reduced in both the GFP single-positive and GFP^+^mCherry^+^ double-positive cells, indicating that RSV infection was suppressed in both the virus-only-infected and virus–bacteria-co-infected populations (Figure 1F; quantified in Figure 1G). Interestingly, we observed a significant increase in the percentage of NTHi+ cells that were co-infected compared to those that were only NTHi-infected, despite there being no significant changes in the MFI of mCherry in either the mCherry single-positive or GFP^+^mCherry^+^ populations (Figure 1H). Because A549 cells are a human lung epithelial cell line, we next sought to validate these observations in a more physiologically relevant system: ex vivo human precision-cut lung slices (hPCLSs). Lung slices were infected with RSV expressing tdTomato (RSV-tdTomato) and NTHi at 10^6^ PFU and 10^7^ CFU, respectively. tdTomato expression was significantly reduced in the RSV–NTHi co-infected samples compared to that in the cells infected with RSV alone at both 24 h and 48 h post-infection, confirming the reduction in RSV in the A549 cells (Figure 1I). Taken together, the results from both the in vitro and ex vivo systems demonstrated that simultaneous RSV–NTHi co-infection suppressed RSV infection. Our data also suggest that such co-infection may enhance the levels of NTHi, especially cell-associated NTHi.

### 3.2. Extracellular NTHi Inhibited RSV Binding to the Host Cell Plasma Membrane

**Figure 2:**
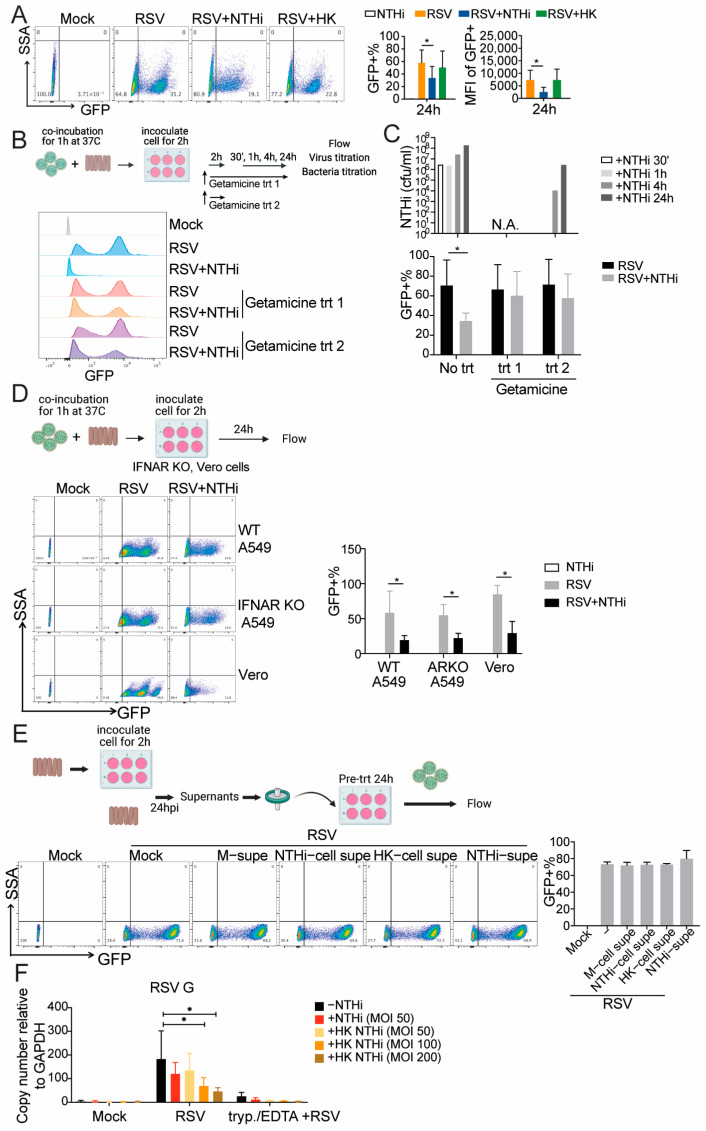
Extracellular NTHi inhibited RSV binding to the host cell plasma membrane. (**A**) A549 cells were co-infected with RSV-GFP with live NTHi or HK-NTHi at a ratio of 1:10, followed by flow cytometry analysis at 24 hpi. N = 3; * *p* < 0.05 according to one-way ANOVA. (**B**,**C**) A549 cells were similarly infected with RSV-GFP and NTHi. Gentamicin (500 μg/mL) was added to the infection medium after the 2 h of inoculation for either 2 h or the remainder of the experiment. GFP+ cells were analyzed by flow cytometry at 24 hpi. Representative flow plots are shown in (**B**) and quantified in (**C**, bottom). Supernatants were titered for NTHi to verify the effects of gentamicin treatment (**C**, upper). N = 3; * *p* < 0.05 according to two-way ANOVA. (**D**) WT A549, IFNAR KO A549, and Vero cells were similarly co-infected with RSV-GFP and NTHi, followed by cytometry analysis at 24 hpi. N = 3; * *p* < 0.05 according to two-way ANOVA. (**E**) Supernatants from mock infection (M-supe), NTHi infection (NTHi-cell supe), HK-NTHi co-infection (HK-cell supe), and NTHi alone (no cells) in the infection medium (NTHi-supe) were collected at 24 hpi, followed by 0.22 filter filtration. Filtered medium was then used to treat fresh A549 cells for 24 h, which were then infected with RSV-GFP at an MOI of 0.5, followed by flow cytometry to monitor GFP+ population at 24 h post-infection. (**F**) A549 cells were inoculated with RSV and live NTHi or increasing amounts of HK-NTHi for 1 h on ice. Cells were washed 3 times with 1xPBS, followed by qPCR targeting RSV G. As a control, incubated cells were first treated with trypsin/EDTA (trypsin/EDTA), followed by 3 washes and qPCR. N = 5 times; * *p* < 0.05 according to one-way ANOVA.

To investigate the mechanisms by which NTHi suppresses RSV during co-infection, we first assessed whether this activity depends on bacterial viability. Heat-killed (HK) NTHi lost the ability to suppress RSV infection, indicating that viable NTHi is necessary to achieve this effect (Figure 2A). Because NTHi can exist both extra- and intracellularly, we next asked whether extra- or intracellular NTHi mediates RSV inhibition. To address this, co-infected cells were treated with gentamicin immediately after inoculation either for 2 h only (trt2) or continuously for the remainder of the experiment (trt 1). Supernatants were collected at 30 min, 1 h, 4 h, and 24 h following the initial 2 h gentamicin treatment, and RSV titers were subsequently assessed at 24 h post-inoculation. Based on bacterial titration of the supernatants, trt2 selectively eliminated extracellular NTHi, while intracellular NTHi was protected and subsequently replicated, leading to its re-emergence in the supernatants from 4 h post-treatment. In contrast, trt1 continuously eliminated extracellular NTHi throughout the experiment (Figure 2C). Notably, both treatments largely restored RSV infection (Figure 2B,C), indicating that extracellular NTHi plays a major role in suppressing RSV infection.

Because RSV infection induces interferon (IFN) responses and RSV is sensitive to type I IFN, we next tested whether IFN signaling contributed to RSV suppression during co-infection. Similar co-infection experiments were performed using IFNAR KO A549 cells and Vero cells, which are type I IFN signaling-deficient. In both cell types, RSV infection was suppressed to a similar extent as in wild-type A549 cells, indicating that RSV inhibition was not mediated by IFN responses. To further test whether extracellular NTHi-derived factors in the supernatant drive RSV suppression, we collected supernatants from cells infected with live NTHi (NTHi-cell supe) or HK-NTHi (HK-cell supe), as well as from NTHi cell-free culture medium (NTHi-supe), followed by filtration to remove bacteria. Fresh A549 cells were pretreated with these filtered supernatants for 24 h prior to RSV infection. No suppressive activity was observed, further supporting a direct role for extracellular NTHi in RSV inhibition (Figure 2E).

Based on these findings, we reasoned that extracellular NTHi inhibits the initial binding of RSV to the host cell plasma membrane. To test this, we performed an RSV binding assay in which A549 cells were co-incubated with RSV and NTHi for 1 h at 4C to prevent viral internalization, followed by extensive washing and collection in Trizol. The amount of RSV particles attached to the cell surface was quantified using qPCR targeting the RSV genome. To enhance the sensitivity of detection of the initial viral input, the RSV MOI was increased to 5 while maintaining an RSV:NTHi ratio of 1:10 for live NTHi. In parallel, RSV (MOI 5) was co-infected with increasing amounts of HK-NTHi. Interestingly, we observed a dose-dependent reduction in the RSV G signal that correlated with the amount of NTHi, regardless of bacterial viability (Figure 2F). To ensure that viral internalization did not occur during the binding assay, infected cells were treated with trypsin/EDTA prior to harvest. Trypsin/EDTA selectively removes virus particles bound to the cell surface, whereas internalized virus particles remain protected within infected cells. This treatment reduced the relative RSV G copy number to near or below the limit of detection, further confirming that NTHi inhibited RSV binding rather than viral internalization (Figure 2F). Taken together, these results suggest that extracellular NTHi must accumulate to a sufficient level to directly inhibit RSV binding to the host cell plasma membrane.

### 3.3. NTHi Significantly Reduced RSV-Induced IFN Responses but Enhanced Pro-Inflammatory Responses During Co-Infection

#### 3.3.1. A549 Cells

**Figure 3:**
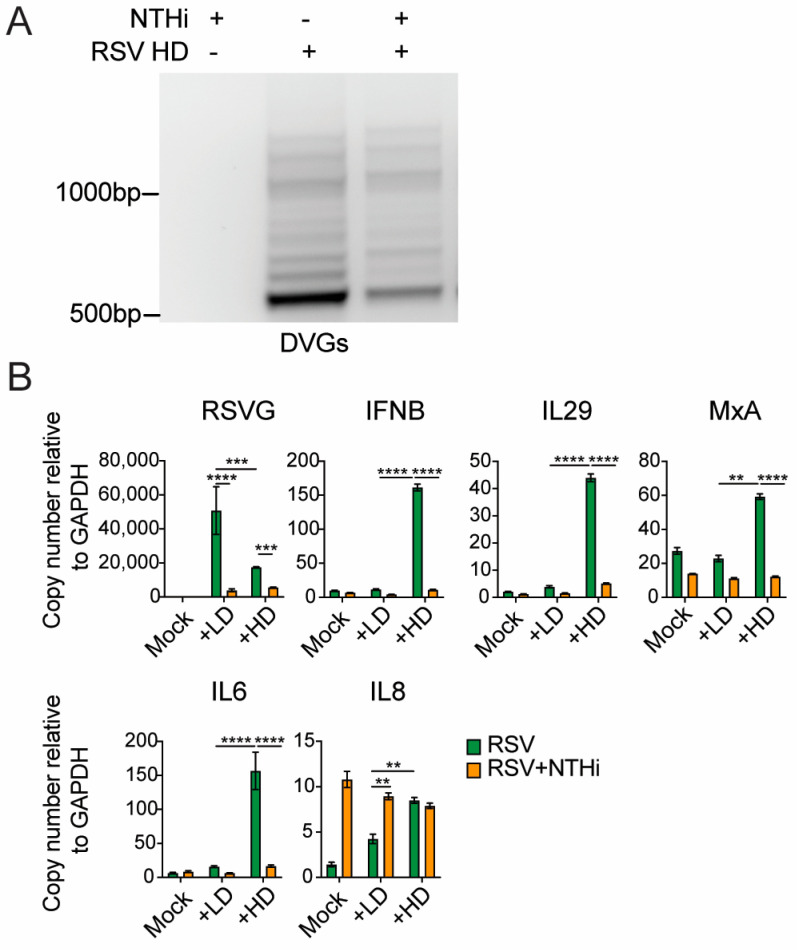
NTHi significantly reduced RSV-induced IFN responses but not pro-inflammatory responses in A549 cells. A549 cells were co-infected with RSV-LD or RSV-HD and NTHi. Cells were harvested at 24 hpi. Total RNA was extracted followed by DVG-specific RT-PCR. (**A**) shows a representative gel image. In parallel, expression levels of IFN-related genes, the pro-inflammatory cytokines IL-6 and IL-8, and representative viral and bacterial genes were quantified by qPCR (**B**). N = 3; ** *p* < 0.01, *** *p* < 0.001, **** *p* < 0.0001 according to two-way ANOVA.

RSV induces robust type I interferon (IFN) responses, and defective viral genomes (DVGs) generated during RSV infection are the primary triggers of these responses [4]. To examine the impact of RSV–NTHi co-infection on RSV-induced host responses, A549 cells were co-infected with NTHi and RSV stocks containing either low (LD) or high (HD) levels of DVGs. We found that, similar to full-length RSV genomes, the amount of DVGs was also reduced by NTHi during co-infection (Figure 3A). As previously reported, RSV-HD induced significantly higher IFN responses than RSV-LD [4]. Consistent with the reduced levels of DVGs, NTHi significantly suppressed IFN responses induced by RSV DVGs. In contrast, the analysis of pro-inflammatory cytokines revealed a different pattern. IL-6 and IL-8 were examined as representative pro-inflammatory mediators. While IL-6 expression showed a trend similar to the IFN responses, the IL-8 levels were not reduced during co-infection compared to those during RSV-LD or RSV-HD infection alone. Notably, IL-8 expression remained comparable between NTHi-only infection and NTHi co-infection with either RSV-LD or RSV-HD (Figure 3B, bottom). Together, these data indicate that NTHi is a relatively stronger inducer of pro-inflammatory cytokines, such as IL-8, than of type I/III IFN responses. Moreover, NTHi inhibited RSV-induced type I IFN responses while preserving pro-inflammatory cytokine production.

#### 3.3.2. hPCLS Model

**Figure 4:**
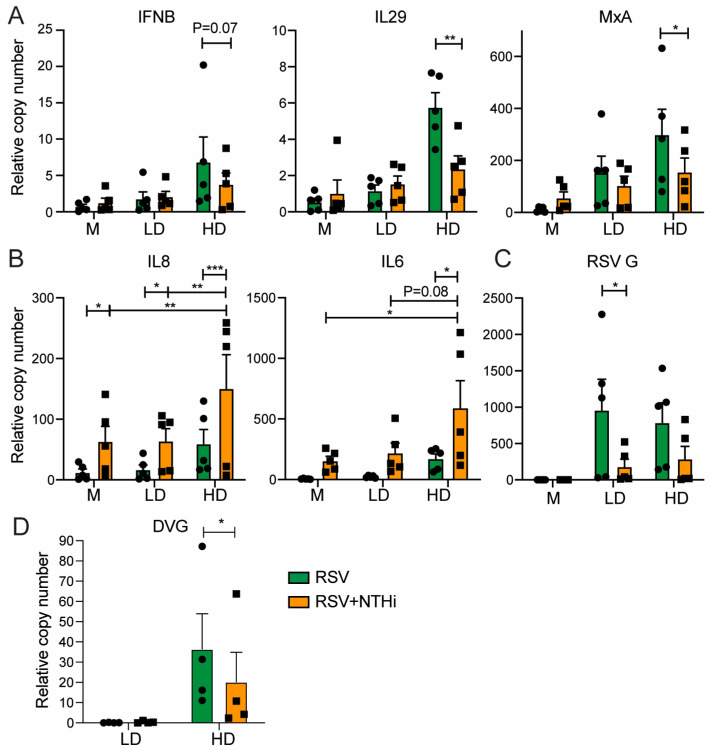
NTHi significantly reduced RSV-induced IFN responses but not pro-inflammatory responses in hPCLSs. hPCLSs were co-infected with RSV-LD or RSV-HD (10^6^ per slice) and NTHi (10^7^ per slice). Slices were harvested at 48 hpi and total RNA was extracted, followed by qPCR to examine the expression levels of IFN-related genes (**A**), the pro-inflammatory cytokines IL-6 and IL-8 (**B**), and RSV G (**C**) and DVGs (**D**). N = 4–5 independent donors. * *p* < 0.05, ** *p* < 0.01, *** *p* < 0.001 according to two-way ANOVA.

**Figure 5:**
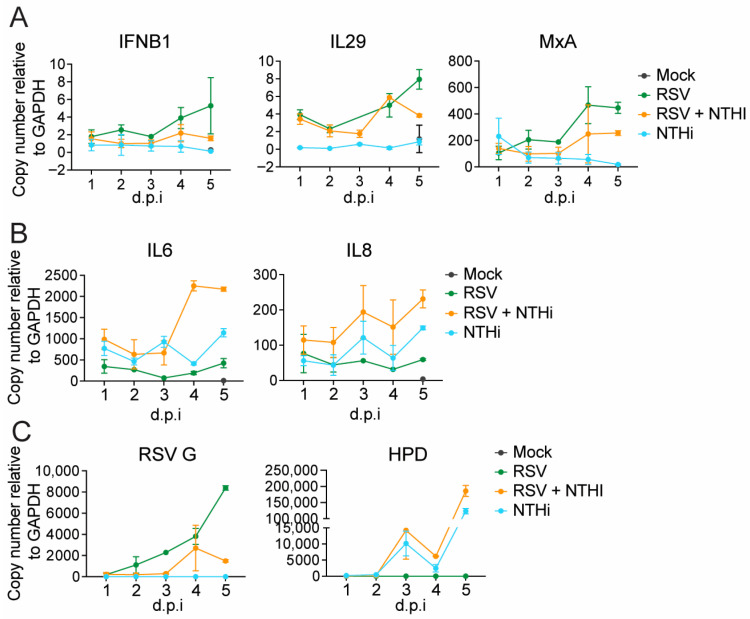
Examination of the kinetics of IFN responses and pro-inflammatory responses in hPCLSs post-RSV-NTHi co-infection. hPCLSs were co-infected with RSV-LD (10^6^ per slice) and NTHi (10^7^ per slice) and slices were harvested each day up to five days post-infection. The expression levels of IFN-related genes (**A**), the pro-inflammatory cytokines IL-6 and IL-8 (**B**), and representative viral and bacterial genes were quantified by qPCR (**C**). N = 2–3 independent donors.

Elevated inflammatory responses have previously been associated with severe RSV disease in patients [2,26]. Therefore, we sought to examine the effect of co-infection on specific host responses in a physiologically relevant model—hPCLSs. hPCLSs were infected using the doses described in Figure 1F, and IFN and pro-inflammatory responses were first assessed at 48 hpi. Compared to single virus infections, co-infection significantly reduced type I IFN responses in the presence of RSV-HD+NTHi, but not RSV-LD+NTHi (Figure 4A). In contrast, compared to RSV-only infections, LD+NTHi co-infections induced pro-inflammatory cytokines, including both IL-6 and IL-8, similar to NTHi-only infection, whereas HD+NTHi co-infection elicited even higher levels of these cytokines than those with NTHi alone (Figure 4B). These data suggest that DVGs can further potentiate NTHi-induced inflammation. As expected, NTHi co-infection was associated with reduced levels of RSV G expression and DVGs (Figure 4C,D). However, we could not reliably detect NTHi HPD gene expression at 48 hpi in hPCLS. To further examine the kinetics of these host responses in hPCLSs, we selected RSV-LD and performed RSV-NTHi co-infections, harvesting slices from day 1 to day 5 post-infection. We consider RSV-LD to more accurately reflect typical clinical scenarios, in which DVGs are generated and accumulated as viral replication peaks, rather than being enriched at the time of initial infection. Similar trends were observed over time. Specifically, type I IFN induction by RSV-LD alone was not distinguishable from the other groups until day 4 post-infection, consistent with previous reports [4]. At later time points, RSV–NTHi co-infection exhibited IFN responses that were lower than those observed with RSV infection alone but higher than those induced by NTHi-only infection (Figure 5A). In contrast, pro-inflammatory responses were highest in RSV–NTHi co-infection—especially at two later time points—intermediate in NTHi-only infection, and lowest in RSV-only infection (Figure 5B). Consistently, NTHi co-infection suppressed RSV G expression, whereas NTHi HPD gene levels remained comparable between NTHi-only infection and RSV–NTHi co-infection, with co-infection again displaying slightly higher levels of HPD (Figure 5C). Notably, HPD gene expression in hPCLS was not detected until D3 post-infection. Together, these findings validated the host response patterns observed in the A549 cells and demonstrated that RSV–NTHi co-infection in hPCLSs led to reduced antiviral IFN responses but enhanced pro-inflammatory signaling at later stages of infection compared to RSV- or NTHi-only infections.

### 3.4. RSV Increased Abundance of Cell-Associated NTHi

**Figure 6:**
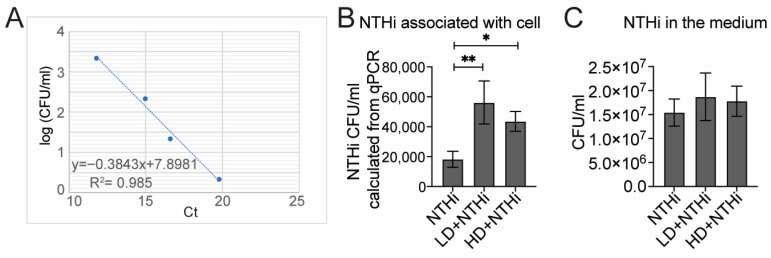
Both RSV LD and HD infections enhanced the cell-associated NTHi. (**A**) NTHi DNA was purified from NTHi cultures with known titers; then, a standard curve of absolute Ct value of HPD gene (from qPCR) and NTHi titer in CFU/mL was developed. (**B**,**C**) A549 cells were firstly infected (or not) with RSV LD and HD (MOI = 0.5) for 6hrs, followed by NTHi infection (MOI = 5) for 40 h. Cells and supernatants were collected at 48 hpi. The genomic DNA of cell-associated NTHi was purified and quantified using HPD gene via qPCR. (**B**) The amount of cell-associated NTHi from co-infection was calculated based on the standard curve presented in (**A**). (**C**) shows the amount of NTHi in the medium. N = 4; * *p* < 0.05, ** *p* < 0.01, according to one-way ANOVA.

It was previously reported that RSV particles can directly bind to NTHi via its glycoprotein to enhance the colonization of NTHi [20]. Additionally, we observed a higher percentage of NTHi-positive cells during co-infection than during NTHi-only infection (Figure 1H). Therefore, we reasoned that the elevated pro-inflammatory responses observed at later time points resulted from increased NTHi associated with RSV-infected cells. To test this, we first established a standard curve of NTHi genomic DNA and its titer to more sensitively quantify cell-associated NTHi. Briefly, we first purified NTHi genomes from four NTHi stocks with known titers. We then performed qPCR to quantify the absolute Ct value of the HPD gene from purified NTHi genomes, and a standard curve was generated of HPD Ct values and their corresponding NTHi titers (Figure 6A). To quantify the cell-associated NTHi, A549 cells were first infected with RSV LD or HD for 6 h, followed by infection with NTHi for 40 h. Infected cells were harvested and NTHi DNA was extracted. The standard curve was then employed to quantify cell-associated NTHi based on the absolute HPD Ct values. We indeed observed a significant increase in NTHi with both LD+NTHi and HD+NTHi than with NTHi alone (Figure 6B). Consistent with Figure 1E, we again observed a nonsignificant trend toward increased NTHi abundance in the supernatants from co-infection (both LD and HD) compared to those from single infections (Figure 6C). Taken together, these data indicated that RSV LD and HD infections enhanced cell-associated NTHi at a similar level, which could contribute to the elevated inflammation observed in hPCLSs infected with RSV+NTHi at later time points (Figure 5).

## 4. Discussion

There are several potential pathways through which patients can harbor viral and bacterial infections: (a) viruses trigger an outgrowth of bacterial pathogens already present within the resident microbiota; (b) viruses promote new bacterial acquisitions as a secondary infection; (c) co-transmission of viruses and bacterial pathogens occurs in respiratory secretions, resulting in simultaneous infection; and (d) prior colonization by specific bacterial species sensitizes young children to RSV infection [27]. For infants with severe disease who are infected with both RSV and NTHi, it remains unclear which of these pathways leads to co-infection. Longitudinal cohort studies of RSV-infected infants have detected NTHi during and, in some cases, prior to RSV infection. In vitro studies further suggest that the chronological order of infection influences RSV infection outcomes: infection with NTHi prior to RSV exposure suppresses RSV infection, whereas NTHi exposure after RSV infection fails to confer this inhibitory effect [20]. To date, few studies have examined the impact of simultaneous RSV–NTHi co-infection, corresponding to scenario (c) above. Our data demonstrated that concurrent co-infection suppressed RSV replication while increasing cell-associated NTHi abundance, and these findings were confirmed in human precision-cut lung slice (hPCLS) models. Collectively, these results suggest that RSV–NTHi co-infection in clinical settings is less likely driven by prior NTHi colonization that predisposes the lung to enhanced RSV susceptibility. Moreover, our findings suggest that the increased disease severity observed in RSV–NTHi co-infected patients is unlikely to result from higher viral burden, but rather arises from dysregulated host immune responses induced by both RSV and enhanced NTHi.

The mechanism by which NTHi suppresses RSV infection was unclear. Under our experimental conditions, we found that extracellular NTHi plays a critical role in RSV inhibition. Specifically, gentamicin treatment, which selectively eliminates extracellular NTHi (trt2), largely restored the proportion of RSV-infected cells (Figure 1C, bottom). However, examination of the flow cytometry plots (Figure 1B) revealed that GFP+ signals in the RSV–NTHi co-infected samples under both trt1 and trt2 remained lower than those observed with RSV infection alone, suggesting that additional mechanisms beyond extracellular NTHi contribute to RSV suppression. Using medium transfer experiments and KO cells, we excluded the possibility that soluble factors secreted by NTHi or host cells mediate RSV inhibition. RSV binding assays directly demonstrated that extracellular NTHi significantly inhibited the binding of RSV particles (~10 × 10^6^) to host cells at >2 × 10^7^ CFU, regardless of whether the bacteria were live or heat-killed. Together, these data indicate that during concurrent co-infection, the initial abundance of NTHi is insufficient to markedly inhibit RSV binding. However, as infection progresses, extracellular NTHi increases exponentially and effectively blocks free RSV particles from binding to naïve host cells. This interpretation is consistent with our flow cytometry data, which showed a strong trend toward RSV inhibition at 16 hpi that became statistically significant by 24 hpi. Furthermore, reduced RSV replication, as reflected by decreased GFP signals, was observed not only in cells co-infected with both pathogens but also in the population infected with only RSV. Previous studies have shown that NTHi can directly bind RSV particles via the RSV glycoprotein G, an interaction that enhances NTHi colonization. It is therefore plausible that this interaction sequesters RSV G and reduces the ability of viral particles to bind to host cells.

Our data derived from A549 cells indicated that RSV infection enhanced NTHi colonization. Although only a modest increase in extracellular NTHi was observed, we consistently detected a significant increase in both the percentage of NTHi-infected cells and the abundance of cell-associated NTHi in co-infected cells compared to cells only infected with NTHi. In agreement with our findings, several studies have reported enhanced NTHi colonization following RSV infection, although the underlying mechanisms vary. For example, NTHi can directly bind RSV particles via the RSV glycoprotein G, which promotes bacterial colonization [20]. In animal models, intranasal RSV infection two days prior to NTHi challenge resulted in a 10–100-fold increase in recoverable NTHi from nasopharyngeal lavage fluids compared to animals infected with NTHi alone. This effect was attributed to reduced expression of chinchilla β-defensin-1 [17]. One limitation of our study was the inability to reliably detect NTHi in hPCLSs, particularly during the early days post-infection, due to technical challenges. To further validate and elucidate the mechanisms by which RSV enhances cell-associated NTHi in hPCLSs, future studies will require immunofluorescence analyses and single-cell RNA sequencing to link specific infected cell types with their corresponding host responses.

Severe RSV disease is associated with delayed type I and type III IFN responses and, later, dysregulated inflammation [2]. One cohort study analyzing nasopharyngeal washes from RSV-positive children indicated that severe RSV disease was associated with increased concentrations of IL-6 and IL-8 [26]. In infants, the presence of NTHi has been associated with the peripheral whole-blood transcriptomic response to primary infection as well as illness severity [6]. Further, the presence of NTHi increases the susceptibility and inflammatory responses of airway epithelial cells in vitro [28]. To examine the host responses, and specifically the innate IFN and pro-inflammatory responses, during simultaneous co-infection, we examined the primary type I and type III IFNs (IFNB1 and IL29), one representative ISG, and two presentative pro-inflammatory cytokines (IL6 and IL8) in infected hPCLSs. hPCLS contains multiple lung-resident cell types, including epithelial cells such as AT1 and AT2 cells, resident macrophages, and dendritic cells, making it a physiologically relevant model of the human lung. However, circulating monocytes are largely removed during tissue preparation, as they are washed out of the lung prior to inflation with low-melting-point agarose and subsequent slicing. We also restricted the included hPCLS donors to children under 5 years of age to better mimic the lungs of infants. First, in single infections, and consistent with previous studies, RSV enriched in DVGs (HD) induced robust IFN responses compared to RSV LD, which was able to induce IFN responses, but only at days 4–5 post-infection. This was likely due to the accumulation of DVGs as viral replication reached high levels at later stages of infection. DVG RNAs are sensed by RIG-I like receptor (RLR), leading to activation of IRF1 and IRF3 and subsequent induction of type I and type III IFNs [4]. Despite Yang et al. showing that NTHi DNAs activate the cGAS-STING-IRF3 pathway to induce type I IFN in macrophages [29], in our study, NTHi infection alone induced a weak IFN response in epithelial cells. Because NTHi strongly inhibited RSV infection including DVG replication, we consistently observed reduced IFN responses in co-infection compared to those in RSV-only infection throughout the course of infection. In contrast to the IFN responses, NTHi alone induced much stronger pro-inflammatory responses. Co-infection with RSV-LD and NTHi induced pro-inflammatory responses comparable to those observed during NTHi-only infection at early time points, which were elevated further at later stages of infection (Figure 5B). Notably, the presence of DVGs further enhanced NTHi-driven inflammation. These host response patterns, together with the enhanced cell-associated NTHi observed in A549 cells, strongly suggest that NTHi exacerbates RSV disease severity by delaying early antiviral IFN responses while amplifying inflammatory signaling at later stages of infection, when viral replication peaks.

It has been reported that lipooligosaccharide (LOS) and the protein P6 on the outer membrane of NTHi stimulate TLR2 and TLR4, activate NF-κB, and promote transcription of pro-inflammatory cytokines, such as IL-6 and IL-8 (reviewed in [30]). Moreover, RSV G proteins interact with TLR2 [31], whereas RSV F proteins interact with TLR4 [32], both contributing to IL-6 and IL-8 production. In the context of co-infection, we hypothesize that RSV G and F proteins, together with RSV DVG RNAs and NTHi LOS and P6, synergistically stimulate TLR2, TLR4, and RLR signaling pathways. This coordinated activation may enhance IRF3 and NF-κB signaling, thereby further elevating pro-inflammatory cytokine production. In addition, extracellular vesicles (EVs) derived from the outer membrane of Gram-negative bacteria are reported to contain immunostimulatory components, including lipopolysaccharide and various outer membrane proteins (reviewed in [33]). Studies have shown that incubating respiratory epithelial cells with purified NTHi EVs induces IL-8 release [33]. Another study revealed that exposing THP-1 macrophages to NTHi EVs triggers the release of IL-8, IL-1β, and THF-α [34]. Further in vivo studies and deep sequencing analyses will be required to define the underlying molecular mechanisms of enhanced inflammation during co-infection.

Other than NTHi, another bacterial species of the nasopharyngeal microbiota that has been reported to be associated with RSV disease severity is Streptococcus pneumoniae [35,36]. Mechanistic studies have shown that both NTHi and S. pneumoniae directly interact with RSV via the RSV G protein, which enhances the colonization of both bacterial species in respiratory epithelial cells [20]. Thus, it is likely that S. pneumoniae also inhibits RSV binding to host cells by sequestering viral particles during co-infection. Regarding host responses, it was previously suggested that RSV and S. pneumoniae co-infection results in an increase in the production of pro-inflammatory cytokines, such as IL-8 and TNFα, in ciliated epithelium [37]. Likewise, RSV can modify the composition of microbiota. For example, it promotes the outgrowth of *S. pneumoniae* and *M. Catarrhalis* [27,36] while being associated with the loss of *Veillonella* and *Staphylococcus* [12]. These observations highlight the broader impact of RSV on the respiratory microbial ecosystem beyond its interaction with NTHi. Therefore, it will be equally important to investigate how RSV interacts with other clinically relevant bacterial species and how such co-infections shape host antiviral and inflammatory responses to influence disease outcomes. In summary, our studies demonstrate that simultaneous RSV–NTHi co-infection suppresses RSV binding and replication while increasing cell-associated NTHi. Correspondingly, co-infection reduces innate IFN responses while amplifying pro-inflammatory signaling. Our findings provide mechanistic insight into how bacterial–viral interactions drive dysregulated innate immune responses that contribute to RSV disease severity, underscoring the importance of the microbial context in shaping RSV pathogenesis and therapeutic strategies.

## Figures and Tables

**Table 1:** List of primer sequences.

Primer Name	Primer Sequences
GAPDH-F	5′-GCAAATTCCATGGCACCGT-3′
GAPDH-R	5′-TCGCCCCACTTGATTTTGG-3′
IFNB-F	5′-GTCAGAGTGGAAATCCTAAG-3′
IFNB-R	5′-ACAGCATCTGCTGGTTGAAG-3′
IL29-F	5′-CGCCTTGGAAGAGTCACTCA-3′
IL29-R	5′-GAAGCCTCAGGTCCCAATTC-3′
MxA-F	5′-GGCTGTTTACCAGACTCCGACA-3′
MxA-R	5′-CACAAAGCCTGGCAGCTCTCTA-3′
IL6-F	5′-GCCCAGCTATGAACTCCTTCT-3′
IL6-R	5′-GCGGCTACATCTTTGGAATCT-3′
IL8-F	5′-GAGCACTCCATAAGGCACAAA-3′
IL8-R	5′-ATGGTTCCTTCCGGTGGT-3′
RSVDI1-F	5′-CTTAGGTAAGGATATGTAGATTCTACC-3′
gRSVDI-R	5′-GTGTCAAAAACTAATATCTCGT-3′
RSVG-F	5′-AACATACCTGACCCAGAATC-3′
RSVG-R	5′-GGTCTTGACTGTTGTAGATTGCA-3′
DVG-F	5′-CGAGAAAAAAAGTGTCAAAAACTAATATC-3′
DVG-R	5′-TATTCGCAGGACCTATTGTAAGG-3′
NHTi-HPD-F	5′-GGTTAAATATGCCGATGGTGTTG-3′
NHTi-HPD-R	5′-TGCATCTTTACGCACGGTGTA-3′

## Data Availability

The original contributions presented in this study are included in the article. Further inquiries can be directed to the corresponding author.

## Abbreviations

The following abbreviations are used in this manuscript:

MFI	Mean fluorescence intensity
MOI	Multiplicity of infection
RSV	Respiratory syncytial virus
NTHi	Nontypeable *Haemophilus influenzae*
HPD	Protein D
HK	Heat-killed
DVGs	Defective viral genomes
RSV-LD	RSV stock with low content of DVGs
RSV-HD	RSV stock with high content of DVGs
PFU	Plaque-forming unit
CFU	Colony-forming unit
hPCLS	Human precision-cut lung slice
IFN	Interferon
EDTA	Ethylenediaminetetraaccetic acid
hpi	Hours post-infection
